# DJ-1 can inhibit microtubule associated protein 1 B formed aggregates

**DOI:** 10.1186/1750-1326-6-38

**Published:** 2011-06-06

**Authors:** Zhiquan Wang, Yu Zhang, Shi Zhang, Qianqian Guo, Yuyan Tan, Xinyi Wang, Ran Xiong, Jianqing Ding, Shengdi Chen

**Affiliations:** 1 Laboratory of Neurodegenerative Diseases & key Laboratory of Stem Cell Biology, Institute of Health Science, Shanghai Institutes for Biological Sciences, Chinese Academy of Science & Shanghai Jiao Tong University School of Medicine. Shanghai 200025, China; 2 Department of Neurology & Institute of Neurology, Ruijin Hospital Affiliated to Shanghai Jiao Tong University School of Medicine. Shanghai 200025, China; 3 Institut Pasteur of Shanghai, Shanghai Institutes for Biological Sciences, Chinese Academy of Science, Shanghai 200025, China

## Abstract

**Background:**

Abnormal accumulation and aggregation of microtubule associated proteins (MAPs) plays an important role in the pathogenesis of neurodegenerative diseases. Loss-of-function mutation of DJ-1/Park7 can cause early onset of PD. DJ-1, a molecular chaperone, can inhibit α-synuclein aggregation. Currently, little is known whether or not loss of function of DJ-1 contributes to abnormal MAPs aggregation in neurodegenerative disorders such as PD.

**Results:**

We presented evidence that DJ-1 could bind to microtubule associated protein1b Light Chain (MAP1b-LC). Overexpression of DJ-1 prevented MAP1b-LC aggregation in HEK293t and SH-SY5Y cells while DJ-1 knocking down (KD) enhanced MAP1b-LC aggregation in SH-SY5Y cells. The increase in insoluble MAP1b-LC was also observed in the DJ-1 null mice brain. Moreover, in the DJ-1 KD SH-SY5Y cells, overexpression of MAP1B-LC led to endoplasmic reticulum (ER) stress-induced apoptosis.

**Conclusion:**

Our results suggest that DJ-1 acts as a molecular chaperone to inhibit MAP1B aggregation thus leading to neuronal apoptosis. Our study provides a novel insight into the mechanisms that underly the pathogenesis of Parkinson's disease (PD).

## Backgrounds

PD is a common neurodegenerative disease which affects approximately 1% of individuals of 65 years and 5% of those 85 years or older. The featured pathological changes of PD are the selective and progressive loss of dopaminergic (DA) neurons as well as protein aggregation and Lewy body formation [1,2]. Lewy bodies mainly constitute of aggregated α-synuclein protein and they also contain cytoskeletal components and other proteins. Although the role of protein aggregation in the pathogenesis of neurodegenerative diseases remains controversial, many studies have shown that protein aggregation contributes to neurodegeneration [2-5]. Failure to clear misfolded proteins leads to protein aggregation, which may in turn lead to the pathogenesis of neurodegenerative diseases.

It has been reported that cytoskeletal proteins are involved in the pathology of neurodegenerative diseases [6,7]. For example, tau has been linked to both Alzheimer's disease (AD) and PD [8,9]. MAP1b has also been reported to participate in the pathogenesis of Fragile X syndrome [10] and Giant axonal neuropathy [11]. MAP1b plays a principal role in the development of the nervous system and is essential for normal development of the murine nervous system [12,13]. It has been reported that MAP1b co-localized with α-synuclein in the Lewy body [14], which provides a hint that insoluble MAP1b may contribute to the pathogenesis of PD. Abnormal accumulation of MAP1B-LC leads to neuronal death in Giant Axonal Neuropathy (GAN) knockout (KO) mice [11]. So it is important to explore whether there is any link between MAP1b aggregation and PD pathogenesis.

Loss of function mutation of Park7/DJ-1 contributed to the pathogenesis of early-onset Parkinsonism [15]. Several PD-causing mutations have been identified including exon deletions, truncations, homozygous and heterozygous point mutations, which are all predominantly in the loss of function manner [16]. DJ-1 belongs to the ThiJ/PfpI superfamily and expresses in both neurons and astrocytes [17,18]. DJ-1 could function as a molecular chaperone [18,19] and inhibit the aggregation of α-synuclein [20,21]. However, the exact role of DJ-1 in the cytotoxic process induced by MAPs aggregation is poorly understood. Here we reported that DJ-1 could directly bind to MAP1b-LC and inhibit its aggregation. Aggregation of MAP1b-LC was exacerbated when DJ-1 was deficient. Furthermore, we also showed that the excessive aggregation of MAP1b-LC could lead to apoptosis in DJ-1 KD SH-SY5Y cells. Therefore, DJ-1 may act as a molecular chaperone to suppress the neuronal death caused by protein aggregation.

## Results

### DJ-1 interacted with MAP1b-LC

**Figure 1:**
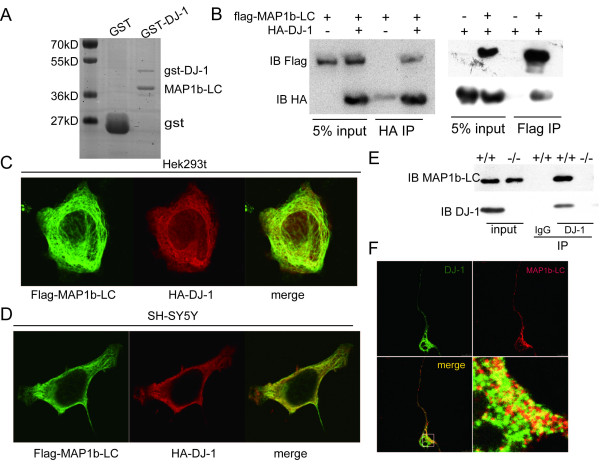
**DJ-1 interacted with MAP1b light chain**. A, GST-DJ-1 or GST was incubated with 6xhis-MAP1b-LC for 3 hrs and then was pulled down by GST beads, the beads were washed and SDS-PAGE followed by Coomassie blue staining was used to analyze the result. The result showed DJ-1 could bind to MAP1b-LC directly *in vitro*. B, Flag-MAP1b-LC and HA-DJ-1 were transfected into HEK293t cells for 36 hrs and the cell lysates were immunoprecipitated with Flag M2 beads or HA antibody conjugated beads. Both DJ-1 and MAP1b-LC were detected by western blot with HA or Flag antibody respectively. C and D, Co-localization of MAP1b-LC and DJ-1. Flag-MAP1b-LC and HA-DJ-1 were cotransfected into KEK293t (C) or SH-SY5Y cells (D) for 36 hrs before fixed and stained with rabbit anti-Flag and mouse anti-HA antibodies. E. The wild type or DJ-1 KO mouse brain lysates were immunoprecipitated with DJ-1 antibody, MAP1b-LC could be detected in the wild type mouse brain but not in that of the KO mouse. F, The primary cortical neuron was fixed and immuno-stained with DJ-1 and MAP1b-LC antibody.

MAP1b-LC has been shown to be a potential DJ-1 binding protein [22]. To study whether DJ-1 can interact with MAP1b-LC, GST-DJ-1 fusion protein and 6xHis-MAP1b-LC were expressed in E. coli BL21 cells and purified respectively. The pull down assay showed that MAP1b-LC interacted with GST-DJ-1 but not with GST, suggesting that DJ-1 could bind to MAP1b directly in vitro (Figure 1A). Flag tagged MAP1b-LC and HA tagged DJ-1 were co-transfected into HEK293T cells for 36 hours. Cells were lysed and immunoprecipitated with either anti-Flag M2 beads or HA antibody-conjugated beads. The results showed that MAP1b-LC and DJ-1 could be immunoprecipitated reciprocally (Figure 1, B). Co-localization of DJ-1 and MAP1b-LC in HEK293t, SH-SY5Y cell lines (Figure 1, C and 1D) and cultured primary neurons (Figure 1F) was also observed. Furthermore, endogenous MAP1b-LC in the wild-type mice brain could also be immunoprecipitated by DJ-1 antibody-conjugated beads (Figure 1, E). These results all demonstrated that DJ-1 could form a complex with MAP1b-LC to regulate the physiological activities of MAP1b-LC.

### DJ-1 could inhibit the aggregation of MAP1b-LC

**Figure 2:**
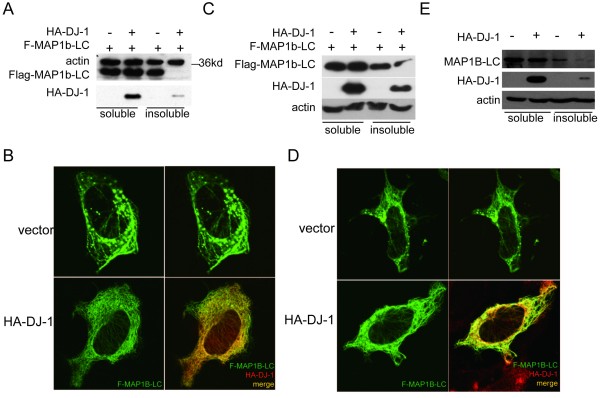
**DJ-1 inhibited MAP1b-LC aggregation in HEK293t and SH-SY5Y cells**. A, Flag-MAP1b-LC was co-transfected with HA-DJ-1 or pcDNA3 vector into HEK293t cells for 48 hrs before the cells were lysed and separated into Triton x-100 soluble and insoluble fractions. Western blot results showed overexpression of DJ-1 almost abolished the insoluble Flag-MAP1b-LC. B, Flag-MAP1b-LC and HA-DJ-1 or pcDNA3 were co-transfected into HEK293t cells for 48 hrs before the cells were fixed and immuno-stained with rabbit anti-Flag or mouse anti-HA antibodies. The most representative images were shown and the results showed the inhibition of MAP1b-LC aggregates in the HA-DJ-1 transfected cells. C, Flag-MAP1b-LC was co-transfected with pcDNA3-HA-DJ-1or pcDNA3 into the SH-SY5Y cells and cultured for 48 hrs before the cells were lysed and separated into Triton X-100 soluble and insoluble fractions. Western blot results showed that overexpression of DJ-1 suppressed the insoluble Flag-MAP1b-LC. D, SH-SY5Y cells were transfected with Flag-MAP1b-LC and pcDNA3-HA-DJ-1 or pcDNA3 respectively. Forty-eight hours later the cells were fixed and stained with rabbit anti-Flag or mouse anti-HA. The results showed the inhibition of MAP1b-LC formed aggregates in the HA-DJ-1 transfected cells. E, pcDNA3-HA-DJ-1 or pcDNA3 was transfected into the SH-SY5Y cells for 48 hrs before the cells were lysed and separated into Triton x-100 soluble and insoluble fractions. Western blot results suggested overexpression of DJ-1 could suppress the endogenous insoluble MAP1b-LC.

It has been reported that MAP1b-LC is a component of cortical Lewy bodies [14], and abnormal accumulation of MAP1B-LC in the animal model of GAN could lead to neuronal death [11]. DJ-1 is thought to be a molecular chaperone that can inhibit the aggregation of α-synuclein [20,21]. To explore whether DJ-1 can affect the aggregation of MAP1b, The Flag tagged MAP1b-LC construct was transfected into the HEK293t cells for 48 hrs. Cells were lysed and the lysates were separated into the detergent soluble and insoluble fractions [20]. These fractions were analyzed by SDS-PAGE/immunoblotting. The results showed that MAP1b-LC formed aggregates were in the insoluble fraction (Figure 2A) and overexpressed DJ-1 decreased the insoluble MAP1b-LC (Figure 2A).

To further evaluate the effect of DJ-1 on MAP1b-LC aggregation, MAP1b-LC construct was transfected into HEK293t cells with either DJ-1 or the empty pcDNA3 vector as a control. We observed less MAP1b-LC aggregates in the DJ-1 overexpressed cells compared with the control (Figure 2B).

The relationship between DJ-1 and MAP1B-LC aggregation was also confirmed in the dopaminergic SH-SY5Y cells. Overexpression of DJ-1 decreased the insoluble MAP1b-LC fraction and inhibited the formation of MAP1b-LC aggregates when they were co-transfected into SH-SY5Y cells (Figure 2 C, D). Moreover, overexpressed DJ-1 also decreased endogenous insoluble MAP1b-LC in SH-SY5Y cells (Figure 2 E). Taken together, these data revealed that DJ-1 could act as a chaperone to inhibit the abnormal aggregation of MAP1b-LC in both HEK293t cells and SH-SY5Y cells.

### Malfunction of DJ-1 exacerbated aggregation of MAP1b-LC

**Figure 3:**
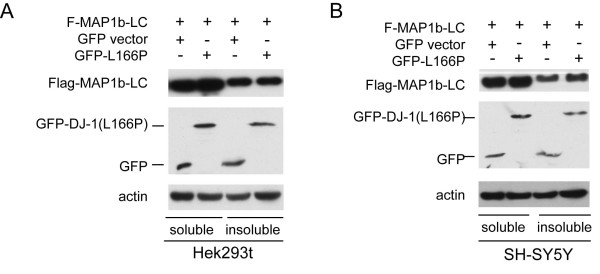
**L166P mutation of DJ-1 decreased the ability of DJ-1 to inhibit the aggregation of MAP1b-LC**. A MAP1b-LC was co-transfected with EGFP-DJ-1L166P or pEGFP-C into HEK293t cells for 48 hrs. Subsequently the cells were lysed and separated into Triton X-100 soluble and insoluble fractions. Western blot results showed that DJ-1L166P could not decrease the level of insoluble Flag-MAP1b-LC. B, MAP1b-LC was co-transfected with pEGFP-DJ-1L166P or pEGFP-C vector into SH-SY5Y cells for 48 hrs. Subsequently the cells were lysed and separated into Triton X-100 soluble and insoluble fractions. Western blot results showed that DJ-1L166P could not decrease the level of insoluble Flag-MAP1b-LC.

**Figure 4:**
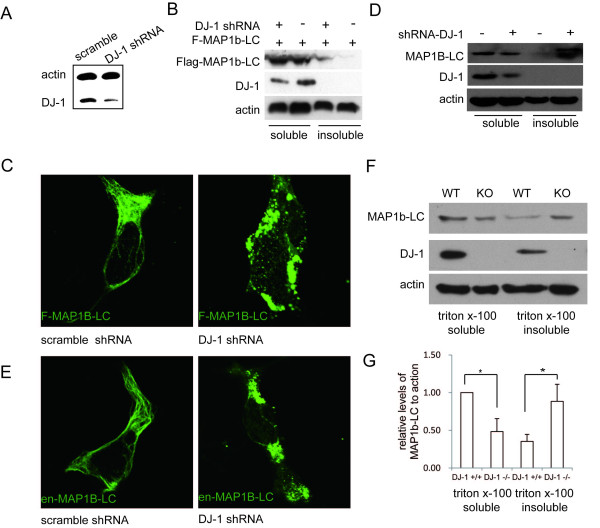
**Loss of function of DJ-1 increased the aggregation of MAP1b-LC**. A, DJ-1 shRNA vector or scramble shRNA vector was transfected into SH-SY5Y cells and selected with 300 μg hygromycin. The stable clone was picked and amplified. Western blot result showed efficient down-regulation of DJ-1. B, Flag-MAP1b-LC was transfected into DJ-1 shRNA or scramble SH-SY5Y cells for 48 hrs. Cells were lysed and separated into Triton X-100 soluble or insoluble components. The result showed increased insoluble MAP1b-LC in the DJ-1 KD cells. C, Flag-MAP1b-LC was transfected into DJ-1 shRNA SH-SY5Y or scramble shRNA cells for 48 hrs. Cells were immuno-stained with rabbit anti-Flag antibody. The results showed the enhancement of MAP1b-LC formed aggregates in DJ-1 KD cells. D, The total lysates of DJ-1 KD SH-SY5Y cells or scramble controls were separated into Triton X-100 soluble or insoluble fractions. Increased insoluble MAP1b-LC in DJ-1 KD cells was observed by Western blot. E, Endogenous MAP1b-LC was examined by immunofluorescence assay in DJ-1 KD SH-SY5Y cells or scramble controls. The MAP1b-LC formed aggregates increased in DJ-1 KD cells. F and G, DJ-1 deficiency intensified the formation of insoluble MAP1b-LC *in vivo*. F, The wild type or DJ-1 KO mice brain lysates were separated into Triton X-100 soluble or insoluble fractions. Western blot was used to analysis of MAP1b-LC distribution in the soluble or insoluble fractions. G, Quantification of relative levels of MAP1b-LC (*, p = 0.03).

L166P, the most common form of DJ-1 mutation, can prevent the dimer formation. The DJ-1 mutant is unstable and is degraded rapidly [23]. These observations have suggested that the DJ-1 mutation could be a loss-of-function mutation. To examine whether or not the DJ-1 mutation impairs the ability of DJ-1 to inhibit MAP1B-LC aggregation, Flag-MAP1b-LC was co-transfected into HEK293t or SH-SY5Y cells with either pEGFP-DJ-1 L166P or pEGFP vector. The results showed that L166P mutation of DJ-1 failed to suppress the accumulation of insoluble MAP1b-LC (Figure 3 A, B). DJ-1 KD SH-SY5Y cell line was also established to further investigate whether the malfunction of DJ-1 could induce the aggregation of MAP1b-LC. Western blot results confirmed that DJ-1 was effectively knocked down in the DJ-1 shRNA stable cells compared with scramble shRNA control (Figure 4A). To examine the effect of down regulation of DJ-1 on the aggregation of MAP1b-LC, the Flag-MAP1b-LC was transfected into DJ-1 KD cells or scrambled control cells. The result showed that there was more insoluble Flag-MAP1b-LC in the DJ-1 KD cells compared with the controls (Figure 4B). Similarly, increased Flag-MAP1b-LC aggregation was also observed in the DJ-1 KD cell lines (Figure 4C). Furthermore, we also observed the increased endogenous insoluble MAP1b-LC (Figure 4D) and the endogenous MAP1b-LC aggregates was observed in DJ-1 KD SH-SY5Y cells (Figure 4E).

The relationship between MAP1b-LC aggregation and DJ-1 deficiency was also studied *in vivo*. Six-month-old DJ-1 KO mice or wild type littermates were used. The brain lysates of 3 KO or wild type mice were extracted and separated into Triton-X100 soluble and insoluble components. The Western blot results showed an increase in insoluble MAP1b-LC in the DJ-1 KO mouse compared with that of the wild type (Figure 4F, G). Taken together, our results showed that DJ-1 abolishment enhanced MAP1b-LC aggregation both in vitro and in vivo.

### DJ-1 abolishment did not alter the ubiquitination of MAP1b-LC and the activity of proteasome

**Figure 5:**
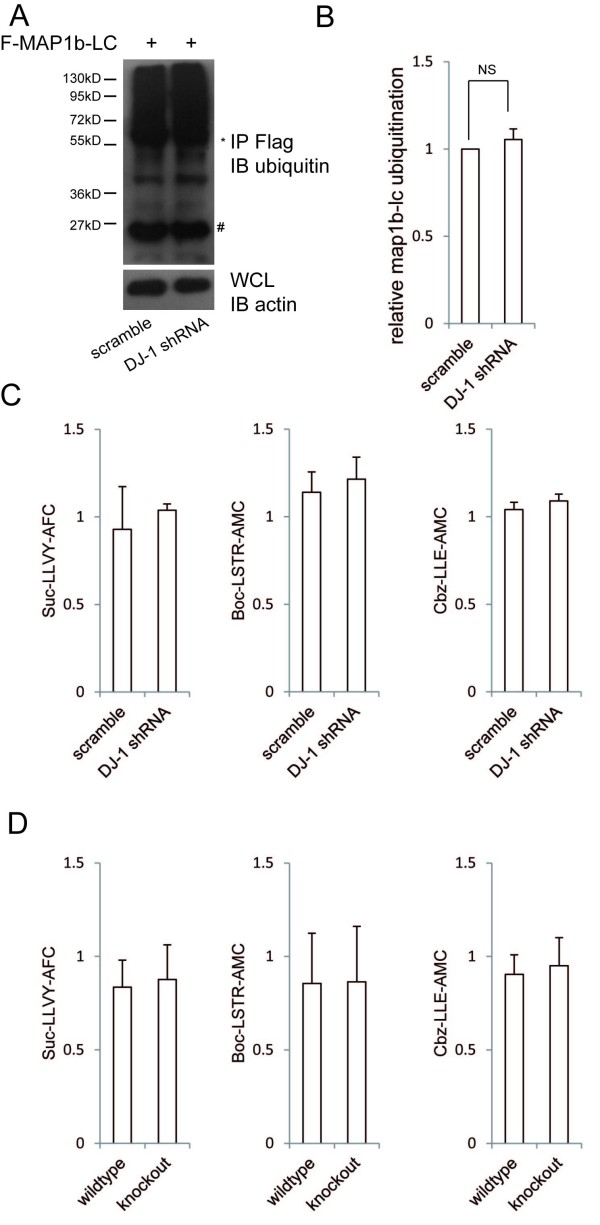
**DJ-1 deficiency did not affect MAP1b-LC ubiquitination and functions of ubiquitin proteasomes**. A, B Flag-MAP1b-LC was transfected into DJ-1 KD SH-SY5Y cells or scramble controls for 48 hours. The Flag-MAP1b-LC was immunoprecipated and immunoprobed with ubiquitin antibody. The asterisk indicated IgG heavy chain and "#" indicated IgG light chain. C, the proteasome activity of scramble and DJ-1 KD SH-SY5Y cells was measured by the fluorescent peptides succinyl-Leu-Leu-Val-Tyr-AFC, Z-Leu-Leu-Glu-AMC, or Boc-Leu-Arg-Arg-AMC, respectively. D, the proteasome activity of wild type (n = 9) and DJ-1 KO (n = 5) mice brain was measured.

MAP1b-LC is degraded through the ubiquitin proteasome system (UPS) and impairments of the UPS in the GAN-null mice may lead to the accumulation of MAP1b-LC [11]. DJ-1 has also been shown to form a complex with Pink1 and Parkin to promote degradation of unfolded or misfolded proteins [24]. Since failure of UPS has been thought to play a critical role in the pathogenesis of PD, we explored whether ubiquitination of MAP1b-LC was altered in the DJ-1 KD cells. Flag-tagged MAP1b-LC was transfected into the DJ-1 KD SH-SY5Y cells or scrambled control cells. Cells were lysed and the lysates were immunoprecipated with anti-Flag antibody and probed with anti-ubiquitin antibody. The result showed that the ubiquitination of MAP1b-LC was unchanged when DJ-1 was knocked down (Figure 5A and 5B).

Down-regulation of DJ-1 can enhance the death of proteasome inhibitor-treated Neuro2A cells [25], so we attempted to assess whether DJ-1 deficiency may affect the proteasome activity. Proteasome chymotrypsin-like, caspase-like and trypsin-like activities were measured with succinyl-Leu-Leu-Val-Tyr-AFC, Z-Leu-Leu-Glu-AMC and Boc-Leu-Arg-Arg- AMC, respectively [26]. The results did not show any significant difference between the proteasome activity of DJ-1 KD cells and that of scrambled control (Figure 5C). Using the same method, we did not find the impairment of proteasome activity in DJ-1 KO mice brain either (Figure 5D). These results suggest that the DJ-1 deficiency did not affect the activity of UPS and ubiquitination of MAP1b-LC. DJ-1 may work as a molecular chaperone to regulate the folding of MAP1b-LC but not its ubiquitination.

### MAP1b-LC aggregation caused ER stress dependent apoptosis in the DJ-1 KD SH-SY5Y cells

**Figure 6:**
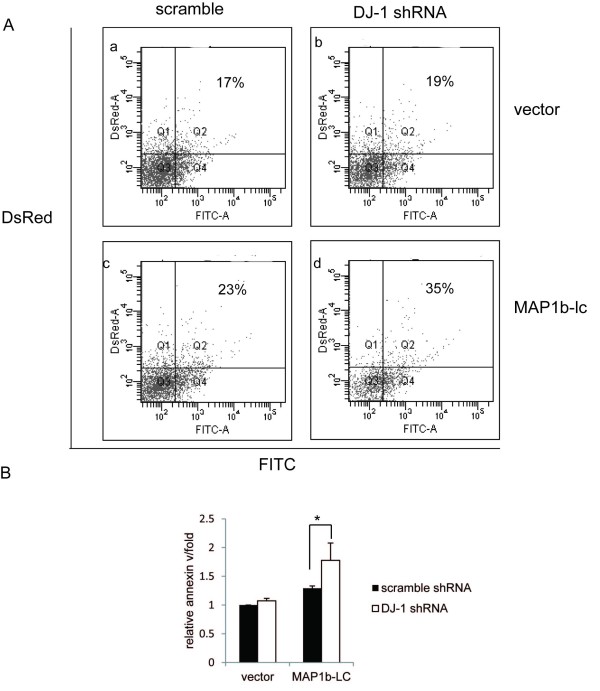
**overburdened aggregation of MAP1b-LC in the DJ-1 KD SH-SY5Y cells leads to apoptosis**. A, pcNDA3-MAP1b-LC or blank pcDNA3 were transfected into DJ-1 KD SH-SY5Y cells or scrambled controls. pDsRed2 was co-transfected to mark the transfected cells. Sixty hours later, cells were stained by Annexin-v and analyzed by flow cytometer. The data showed that MAP1b-LC can induce apoptosis (23% Annexin-v positive of the total transfected cells); the apoptosis cells increased to 35% in the DJ-1 KD cells compared to the scramble. B, The statistical analysis result showed that the apoptosis was increased when MAP1b-LC was introduced into DJ-1 KD SH-SY5Y cells (*, p = 0.04)

**Figure 7:**
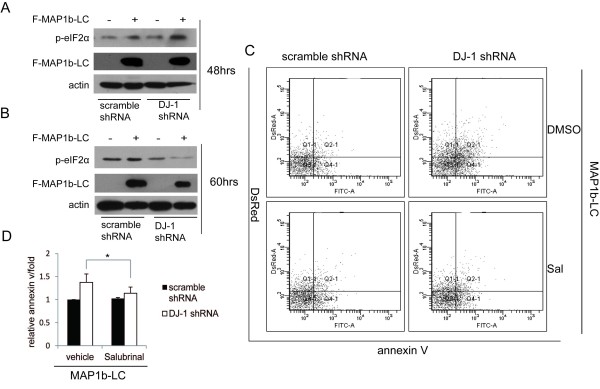
**Apoptosis induced by aggregation of MAP1b-LC in the DJ-1 KD SH-SY5Y was ER stress dependent**. A, An increase in phosphorylated eIF-2α, an early ER stress marker, was observed at 48 hrs after transfection of Flag-MAP1b-LC (F-MAP1b-LC) in the DJ-1 KD SH-SY5Y cells. B, Dephosphorylation of eIF-2α was increased at 60 hrs after transfection of MAP1b-LC in the DJ-1 KD cells. C and D, Salubrinal, inhibitor of eIF-2α dephosphorylation, suppressed the increased apoptosis induced by MAP1b-LC in the DJ-1 KD SH-SY5Y cells (C). Statistic result showed Salubrinal inhibited the increased apoptosis in the DJ-1 KD SH-SY5Y cells induced by MAP1b-LC (D) (*, p = 0.016).

Since protein aggregation is a major cause of neurodegeneration and malfunction of DJ-1 can lead cells to produce more aggregated MAP1b-LC, we next studied whether MAP1b-LC aggregation induced apoptosis of DJ-1 KD SH-SY5Y cells. We transfected the MAP1b-LC into DJ-1 KD SH-SY5Y cells or scrambled control, pDsRed2 being co-transfected with MAP1b-LC for the selection of the transfected cells. After 60 hours, the cells were harvested for the Annexin-V apoptosis assay. We observed more Annexin-V positive cells in the MAP1b-LC transfected group (Figure 6A, d) compared to that of the scramble cells (Figure 6A, c) (Figure 6 B). Since apoptosis caused by protein aggregation is mainly through the ER stress dependent pathway [27], we examined the phosphorylated eIF2α, an ER stress marker. Phosphorylation of eIF2α will only happen at the early stage of ER stress to counteract the insult and is thought to be protective for the cells from ER stress. However, when the insult continues, activated eIF2α will be dephosphorylated and the protection will be abolished, leaving the cells to undergo apoptosis [28,29]. Our results showed that phosphorylated eIF2α was much higher in MAP1b-transfected DJ-1 KD cells at 48 hrs after transfection compared with that in the scrambled control DJ-1 KD cells (Figure 7A). However, eIF2α dephosphorylation in the MAP1b-LC transfected DJ-1 KD cells was increased at 60 hrs after transfection (Figure 7B). These results showed that phosphorylation of eIF2α was induced upon MAP1b-LC aggregation to protect the cells against ER stress and increased MAP1b-LC aggregation induced severer ER stress in DJ-1 KD cells. However, the protection was abolished as the DJ-1 deficient cells failed to decrease the aggregated proteins in the DJ-1 KD cells, which finally led to ER stress induced apoptosis. Particularly, Salubrinal, the specific inhibitor of dephosphorylation of eIF2α [28] suppressed the MAP1b-LC induced apoptosis in DJ-1 KD SH-SY5Y cells (Figure 7C and 7D). Taken together, these results suggested that excessive MAP1b-LC aggregation caused by DJ-1 ablation may induce apoptosis in the ER stress dependent manner.

## Discussion

Mutations of *DJ-1 *have been linked to early onset Parkinsonism. However, the molecular mechanism underlying the pathogenesis is still obscure. DJ-1 is thought to be a molecular chaperone and oxidative sensor, participating in both familial and sporadic PD [20,30]. Most researches have been focused on the anti-oxidative stress function of DJ-1 [31-35] but the molecular chaperone function of DJ-1 was hardly noticed [20]. Our results showed that DJ-1 interacted with MAP1b-LC both *in vitro and in vivo*. Furthermore, aggregates formed by overexpressed MAP1b-LC in HEK293t and SH-SY5Y cells could be inhibited by the overexpression of DJ-1.

Protein aggregation and insoluble inclusion bodies are one of the main causes for the pathogenesis of many neurodegenerative diseases [2]. MAP1b is essential for normal development of the murine nervous system [13] and its abnormal accumulation has been linked to neurodegenerative disease. Moreover, MAP1b-LC has been observed in the insoluble Lewy bodies in the brain of PD patients [14]. Allen et al. reported that MAP1b-LC accumulation could lead to the neuron death in *GAN *KO mouse [11]. Since DJ-1 has been shown to interact with MAP1b-LC and inhibit its aggregation, we hypothesized that the formation of MAP1b-LC aggregation may be suppressed by the molecular chaperone DJ-1.

Mutations of DJ-1 are thought to be loss function mutations that can lead to autosomal recessive familial PD. The most frequently DJ-1 mutant L166P fails to form dimers and monomeric mutant DJ-1 is unstable and degraded rapidly [23]. Therefore DJ-1 KD SH-SY5Y stable cell lines and DJ-1 KO mice were used to study the effect of DJ-1 on the aggregation of MAP1b-LC. In the DJ-1 KD cells, MAP1b-LC aggregation was increased compared with that of the scramble control cells. Moreover, the level of insoluble MAP1b-LC was also increased in the DJ-1 KO mice. So it implicated that DJ-1 may work as a molecular chaperone to control the normal state of MAP1b-LC and loss function of DJ-1 may lead to increased aggregation of MAP1b-LC.

It has been proposed that the impairments of ubiquitin proteasome system (UPS) play an important role in the pathogenesis of PD. DJ-1 has also been reported to participate in regulating the activity of UPS [24,25]. It has been found that inhibition of MAP1b-LC ubiquitination leads to neuronal death in the GAN-null mice [11]. Therefore, we examined whether increased aggregation of MAP1b-LC results from the failure of its UPS dependent degradation. However, our results did not show any change of the ubiquitination of MAP1b-LC (Figure 5A). Neither was there any change of the total protein (soluble plus insoluble) level of MAP1b-LC (Figure 4 G). Moreover, there was no impairment of proteasome activity in DJ-1 KD cells and DJ-1 KO mice. All of these observations have indicated that the loss-of-function of DJ-1 does not affect the UPS dependent degradation of MAP1b-LC. The increase in insoluble MAP1b-LC in the DJ-1 KD cells and DJ-1 KO mice suggests that DJ-1 may work as a molecular chaperone to promote correct folding of MAP1b-LC or maintain the normal state of MAP1b-LC.

Protein aggregation has been implicated to play an important role in the pathogenesis of neurodegenerative diseases. Therefore, we analyzed whether excessive MAP1b-LC aggregation can cause cell apoptosis. The data suggested that overexpression of MAP1b-LC in the DJ-1 KD SH-SY5Y cells, which produced overburdened MAP1b-LC aggregates, increased cell apoptosis. It has been reported that abnormal protein aggregation can induce apoptosis mainly through the ER stress pathway [36]. Previous studies have implicated that phosphorylation of eIF2α was a protective cell response to counteract the ER stress and the failure of the phosphorylation of elf2α will lead to apoptosis [28,29]. Our results revealed that the enhancement of MAP1b-LC aggregation can induced more phosphorylated eIF2α in DJ-1 KD cells than that in scramble controls. The persistent existence of MAP1b-LC aggregation increased dephosphorylated eIF2α and led the cells to ER stress dependent apoptosis at 60 hrs. The fact that the eIF2α dephosphorylation inhibitor Salubrinal can partially inhibit the apoptosis supported our hypothesis.

DJ-1 has been shown to be a molecular chaperone that can inhibit α-synuclein aggregation [20,21]. However, Ramsey et. al showed that DJ-1-deficient mice had similar vulnerability to pathogenic Ala53Thr human α-synuclein toxicity [37]. Based on our experimental results, we cannot make the conclusion that chaperone activity of DJ-1 is unrelated to alpha-synuclein aggregation. It is possible that compensatory mechanisms exist in DJ-1 null mice which act to mimic the function of DJ-1 protein just as the author of the paper claimed [37].

We observed increased aggregation of MAP1b-LC in DJ-1 KD SH-SY5Y cells, as well as an increased level of insoluble MAP1b-LC in DJ-1 KO mice. In contrast, two previous studies did not observe the formation of inclusion bodies in either adult or aged DJ-1 null mice [38,39]. It suggested that DJ-1 abolishment produced more MAP1b-LC aggregation *in vitro *and more insoluble MAP1b-LC in vivo. There are two potential explanations for the difference between our in vitro and in vivo experiments: 1) In vitro experiment showed the acute responses of the cells to MAP1b overexpression or DJ-1 KD, in contrast, the in vivo study showed the chronic responses of animals to DJ-1 KO; and 2) both environmental and genetic factors are responsible for the pathogenesis of PD. The absence of MAP1b aggregation in DJ-1 KO mice may be due to the absence of certain non-genetic factors such as aging or neurotoxins in our study.

## Conclusions

In summary, we report that DJ-1 is a molecular chaperone that can inhibit the aggregation of MAP1b-LC in vitro as well as the formation of insoluble MAP1b-LC in vivo. Our findings have provided the first evidence that links DJ-1 deficiency to MAPs aggregation, which may improve our understanding regarding the role of DJ-1 in the pathogenesis of PD.

## Materials and methods

### Antibodies and Reagents

The following antibodies were used: DJ-1 Monoclonal Antibody (3E8) (Assay Designs, ADI-KAM-SA100-E), mono- and polyubiquitinylated conjugates, monoclonal Antibody (FK2) (Biomol, BML-PW8810R), DJ-1 polyclonal antibody (Abcam, ab18257), Rabbit polyclonal anti-Flag (F7425), mouse monoclonal anti-Flag (F1804), mouse monoclonal anti-beta-actin (A5441) (Sigma-Aldrich), mouse anti-HA (clone 12C5) (Covance, MMS-101R), mouse anti-EGFP (Roche, 11814460001), Phospho-elF2alpha (Ser51) antibody (Cell Signaling, 9721), Goat polyclonal anti-MAP1b (c-20) (Santa Cruz Biotechnology, sc-8971). EIF-2α inhibitor Salubrinal was purchased from Calbiochem. All the Chemicals were purchased from Sigma-Aldrich except noted elsewhere.

### Plasmid construction

Human DJ-1 and MAP1B-LC cDNA were amplified from the human fetal brain cDNA library (Invitrogen) and ligated to the pcDNA3 vector with an N terminal HA tag and pCMV-3xflag (sigma), respectively. To knock down DJ-1, a DNA fragment and a scramble fragment were synthesized and ligated to the pSilencer-3.1-Hygro (Ambion). The sequence of the inserted DJ-1 DNA fragments is GATCCGCTAAAGGAGCAGAGGAAATTTCAAGAGAATTTCCTCTGCTCCTTTAGTTTTTTGGAAA, and the scramble sequence is GATCCGATCTCTTCTGGTATTAGACTCAAGAGATCTAATACCAGAAGAGATCTTTTTTGGAAA. All the constructions were confirmed by sequencing. PCR-based site directed mutagenesis was used to construct the L166P mutation of DJ-1, which was cloned to the pEGFP-C2 (Clontech).

### Cell culture and transfection

HEK293T and SH-SY5Y cells were purchased from American Type Culture Collection and maintained in DMEM with 10% Fetal Bovine Serum and 100 U/ml penicillin/streptomycin. All the culture materials were purchased from Invitrogen. HEK293t cells were transfected by calcium phosphate precipitation and SH-SY5Y cells were transfected with lipofectamine 2000 (Invitrogen). Transfection efficiency was neutralized by cotransfected with a pRL-tk plasmid. Because DJ-1 L166P is unstable, the amount of the plasmid used for its transfection was 3 times more than that used for the control vector. pSilencer-Hygro-DJ-1 used for DJ-1 KD was transfected into SH-SY5Y and the stable clones were selected with 300ug/ml Hygromycin.

### Co-immunoprecipitation and Western blotting

For co-immunoprecipitation, HEK293T cells or the mice brain were lysed in the buffer A containing 50 mM Tris·HCl pH 7.6, 150 mM NaCl, 0.5% NP-40, 1% sodium deoxycholate and protease inhibitor cocktail (Roche). To detect the ubiquitination of MAP1b-LC, the cells were harvested and boiled in the lysis buffer B (50 mM Tris·HCl pH 7.6, 150 mM NaCl, 1% NP-40, 1% sodium deoxycholate, 1% SDS) for 10 min. Then the concentration of SDS in Buffer B was diluted to 0.1% by RIPA buffer. The lysates were pre-cleared with protein A sepharose (GE bioscience) for 30 min. The supernatants were incubated with the primary antibody for 4 hours at 4°C. Then protein A sepharose (GE Bioscience) was added and the mixture was further incubated for 2 h at 4°C. For the Flag fusion protein IP, Flag M2 beads (Sigma-Aldrich) were used. The beads with bound proteins were washed for 6 times with lysis buffer and were boiled in 2X SDS sample buffer, then the samples were detected by immunoblotting.

For Western blotting the cells were lysed in RIPA buffer (50 mM Tris·HCl pH 7.6, 150 mM NaCl, 1% NP-40, 1% sodium deoxycholate, 0.1% SDS) with protease inhibitor cocktail (Roche). To separate the detergents soluble and insoluble proteins, the homogenized mice brain or the cells were lysed in 0.2% Triton X-100 lysis buffer (25 mM Tris·HCl pH 7.6, 150 mM NaCl, 0.2% Triton X-100, 1% sodium deoxycholate) on ice for 20 min. Triton X-100-soluble and -insoluble fractions were separated via centrifugation at 13,000 rpm for 15 min [20]. The samples were boiled in 2XSDS sample buffer and detected by immunoblotting. For all the western blot results, at least 3 independent experiments were done and the most representative result was shown.

### Immunofluorescence microscopy

HEK293T or SH-SY5Y cells were grown on glass coverslips, fixed with 4% PFA, permeabilized with 0.2% Triton X-100, and blocked with 20% goat serum or 5%BSA in 0.2% PBST and then incubated with primary antibody. Cells were washed and Alexa 594 or Alexa 488 goat anti-mouse or rabbit IgG antibody (Invitrogen) was added. After washed 3 times using PBS, anti-fade mounting medium with DAPI (Vector Laboratory) was added and the stained cells were analyzed with a confocal microscopy (Leica SP5).

### Apoptosis detection by Annexin V assay

Cells were seeded in 6-well plates and transfected with the indicated plasmids and pDsRed2 for 48 hours. Then cells were stained with Annexin V using the Annexin V-FITC apoptosis detection kit (BD bioscience) as the instructions of the manufacturer. Cells (30,000/treatment) were analyzed using a flow cytometer (Becton Dickinson LSR II).

### Animal studies

The DJ-1 knock-out mice were kindly provided from Dr. Jie Shen (Harvard Medical School) [40] and crossed with C57bl/6 mice at least 6 generations after arriving in our lab. Throughout the experiments, the animals were kept in stainless-steel cages in a controlled environment (22-25°C, 40-60% relative humidity, 12-h light-dark cycle), with food and water available freely. All animal experiments were performed in accordance with guidelines of the laboratory animal ethical standards of Shanghai Jiao Tong University School of medicine. For animal studies, in each group 3 littermate mice brains were used, which was defined as one independent experiment. Statistical analyses were conducted on the results from three independent experiments.

### Statistical analysis

Paired or unpaired Student's t-test was used for statistical analyses. Statistical significance was set at a *P *value of less than 0.05 and there was no statistical correction was used for all the values.

## List of Abbreviations

PD: Parkinson's disease; MAP1b LC: microtubule associated protein 1b; ER: endoplasmic reticulum; KD: Knocking down; KO: Knock out; SDS-PAGE: sodium dodecyl sulfate polyacrylamide gel electrophoresis; GST: Glutathione-S-transferase; UPS: ubiquitin proteasome system.

## Competing interests

The authors declare that they have no competing interests.

## Authors' contributions

ZQW designed and performed the experiments. YZ contributed to the protein expression and purification. SZ, XR and XYW helped to perform the animal studies. QQG helped to perform the flow cytometry analysis and YYT provided essential advice to the project. SDC and JQD supervised the project and edited the manuscript. All authors read and approved the final manuscript.
